# Software Tool for Soil Surface Parameters Retrieval from Fully Polarimetric Remotely Sensed SAR Data

**DOI:** 10.3390/s20185085

**Published:** 2020-09-07

**Authors:** Davod Poreh, Antonio Iodice, Antonio Natale, Daniele Riccio

**Affiliations:** 1 Department of Electrical Engineering and Information Technology, University of Napoli Federico II, 80125 Napoli, Italy; iodice@unina.it (A.I.); daniele.riccio@unina.it (D.R.); 2 Institute of Remote Sensing of Environment (IREA), National Research Council (CNR), 80124 Napoli, Italy; natale.a@irea.cnr.it

**Keywords:** polarimetry, PTSM, PTSTCM, PolSAR

## Abstract

The retrieval of soil surface parameters, in particular soil moisture and roughness, based on Synthetic Aperture Radar (SAR) data, has been the subject of a large number of studies, of which results are available in the scientific literature. However, although refined methods based on theoretical/analytical scattering models have been proposed and successfully applied in experimental studies, at the operative level very simple, empirical models with a number of adjustable parameters are usually employed. One of the reasons for this situation is that retrieval methods based on analytical scattering models are not easy to implement and to be employed by non-expert users. Related to this, commercially and freely available software tools for the processing of SAR data, although including routines for basic manipulation of polarimetric SAR data (e.g., coherency and covariance matrix calculation, Pauli decomposition, etc.), do not implement easy-to-use methods for surface parameter retrieval. In order to try to fill this gap, in this paper we present a user-friendly computer program for the retrieval of soil surface parameters from Polarimetric Synthetic Aperture Radar (PolSAR) imageries. The program evaluates soil permittivity, soil moisture and soil roughness based on the theoretical predictions of the electromagnetic scattering provided by the Polarimetric Two-Scale Model (PTSM) and the Polarimetric Two-Scale Two-Component Model (PTSTCM). In particular, nine different retrieval methodologies, whose applicability depends on both the used polarimetric data (dual- or full-pol) and the characteristics of the observed scene (e.g., on its topography and on its vegetation cover), as well as their implementation in the Interactive Data Language (IDL) platform, are discussed. One specific example from Germany’s Demmin test-site is presented in detail, in order to provide a first guide to the use of the tool. Obtained retrieval results are in agreement with what was expected according to the available literature.

## 1. Introduction

Electromagnetic (EM) scattering from the Earth surface depends on a number of parameters (i.e., permittivity, roughness, soil moisture content, vegetation biomass-index, etc.) that characterize the observed scene. Accordingly, large-scale and frequent monitoring of these quantities can be carried out exploiting remote sensing data, on condition that reliable estimation procedures are used [1].

Parameters estimation from remote sensing measurements is, however, everything but an easy task. It indeed requires, on one hand, to recognize and isolate the impact that each parameter has on the measured data and, on the other hand, to build up well-posed estimation procedures, where the number of unknowns does not exceed the available independent measurements (a wider discussion on this issue is reported for instance in [2]).

For this reason, in the last decades a number of retrieval methods based on multi-frequency, multi-angle and/or multi-polarization Synthetic Aperture Radar (SAR) data have been devised to carry out the estimation of quantities that characterize anthropogenic (e.g., the three-dimensional (3D) buildings, or oil slicks) and natural (e.g., snow cover, or vegetation) targets [1,2,3,4,5,6,7,8,9,10,11,12,13,14,15].

In particular, in recent years, some methods for the retrieval of soil surface parameters from polarimetric SAR data have been developed [16,17,18,19]. Among these, model-based retrieval methods rely on the predictions of the polarimetric scattering provided by analytical models. In this regard, the employed model should be sufficiently accurate (i.e., it should realistically describe the scattering surface, and asymptotic evaluations of the scattering integral should be a good approximation of the exact integral in the range of values of surface parameters of practical interest) to provide precise scattering predictions but, at the same time, not too involved (i.e., with a not too heavy computational burden) to be used for the inversion purposes [16,19].

The most popular and comparatively simple analytical solutions for the EM scattering from natural surfaces are probably the Physical-Optics (PO) and the Small Perturbation Method (SPM) [16,17,18,19,20,21]. However, due to their simplicity, these models are not able to take into account the cross-polarization and depolarization effects that are often observed in polarimetric radar data scattered from rough and/or vegetated surfaces. Classical Two-Scale Models (TSM) are able to significantly widen the validity range of such simple models; nevertheless, they do not account for the depolarization effect [21,22,23]. More refined methods, such as the second-order SPM and the Integral Equation Methods (IEM), are instead able to predict the above-mentioned depolarization and cross-polarization effects [2]. However, their formulations do not allow to express the scattered field in a closed form. Accordingly, they are too involved to be used for practical parameters’ inversion purposes [19]. Simpler analytical solutions are instead provided by the recently published Polarimetric Two-Scale Model (PTSM) [19] and Polarimetric Two-Scale Two-Component Model (PTSTCM) [2], which are able to describe the polarimetric properties of the EM field scattered from bare or slightly vegetated soils. A more accurate modeling of the effects of the vegetation layer on the scattered field is provided by the Iterative Generalized Hybrid Decomposition (IGHD) [17,18], which, however, exploits a simplified soil surface scattering model not accounting for depolarization. PTSM, PTSTCM and IGHD provide satisfactory scattering predictions though exhibiting a comparative simplicity. Accordingly, the scattering predictions provided by these models have been used to devise retrieval methods for the estimation of soil permittivity, soil moisture and ground roughness from Polarimetric Synthetic Aperture Radar (PolSAR) data [16,17,18,19]. In particular, recent studies involving actual radar data showed that in low-vegetated areas (i.e., areas characterized by a vegetation height lower than 50 cm and negligible ground-trunks scattering interactions), the PTSTCM-based retrieval method provides better soil parameters estimations with respect to the ones provided by the IGHD (average modulus of soil moisture relative error: 18.5% for the PTSTCM and 34% for the IGHD [2]). Conversely, the IGHD outperforms the PTSTCM in terms of estimation results for significantly vegetated areas (average modulus of relative error: 17.5% for the IGHD and about 100% for the PTSTCM [2]).

In this work we present a novel software package, called PTSM Object-Oriented Program (OOP), for the retrieval of soil surface parameters. Nine different methodologies are implemented in a code developed in the Interactive Data Language (IDL) environment. This user-friendly software provides the necessary algorithms for the estimation of soil surface parameters’ from polarimetric (airborne or satellite) SAR data for novice and expert users. The software package consists of a main widget and many routines to read, prepare, analyze, display and store the output data. The software has been tested in Windows and Linux platforms showing satisfying results. The growing interest in the use of fully polarmetric SAR data, and the absence of this kind of software to calculate reliable soil surface parameters, was the primary motivation. More sophisticated SAR software tools, such as SARscape, ENVI, PolSARPro, Geomatica, Gamma, etc., are designed to deal with SAR/PolSAR data, to retrieve some other important practical parameters such as radar cross sections, coherency and covariance matrixes, Pauli decomposition, etc.; PTSM-OOP is a free software embedded in the IDL environment to estimate soil surface parameters from PolSAR imageries.

The paper is organized as follows: background for implementing this software is explained in Section 2, Section 3 explains the software design and implementation, in Section 4 we illustrate the software functionalities, in Section 5 we present one example and, finally, conclusions are summarized in Section 6.

## 2. Theoretical Background

In this section, we briefly summarize the theoretical frameworks leading to both the PTSM and PTSTCM, which provide, respectively, the solutions for the EM field scattered from bare soil and slightly vegetated soil surfaces. In addition, we recall the PTSM- and PTSTCM-based retrieval methods which can be employed to estimate soil parameters from polarimetric radar data.

The full descriptions of the above-mentioned models and methods can be found in [2,19,23,24,25].

### 2.1. EM Scattering from a Bare Soil Surface: The PTSM

**Figure 1:**
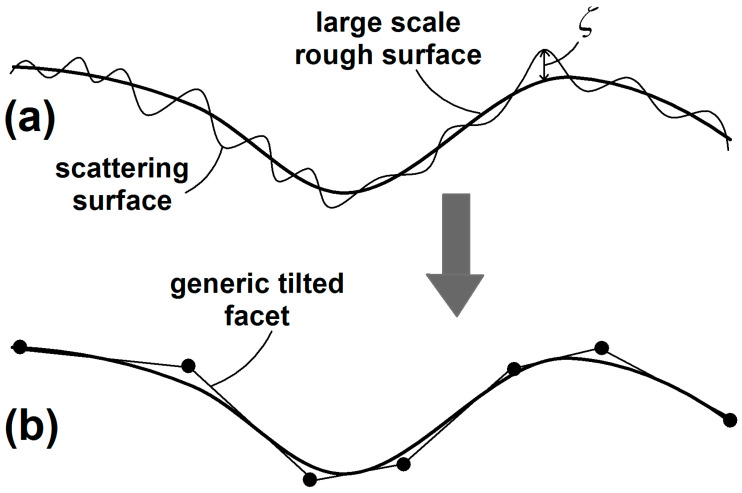
Polarimetric Two-Scale Model (PTSM) surface representation: from actual surface (**a**) to its approximation by facets (**b**).

The scattering surface is considered as composed of large-scale and small-scale roughness. As for the former, it is locally approximated by tangent facets, over which the small-scale roughness is superimposed (see Figure 1). Both the facets’ slopes and the small-scale roughness are described in terms of stochastic processes and, in particular, it is assumed that the SPM or the PO hold for every rough facet.

**Figure 2:**
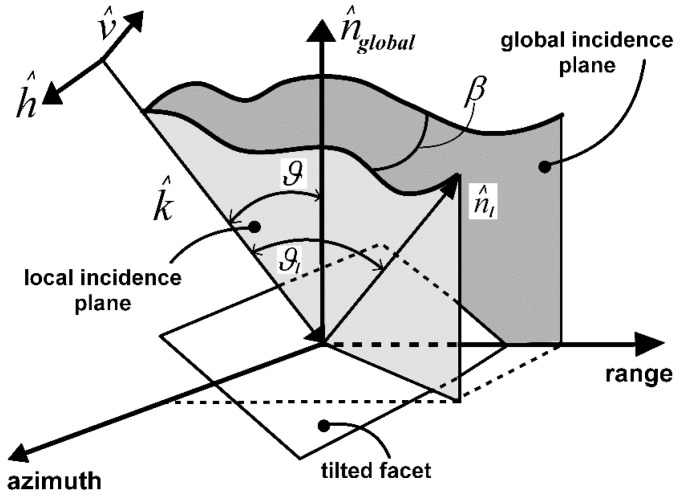
PTSM geometry: local rotation of the incidence plane and drift of the incidence angle.

The facet random tilt causes a random variation of the local incidence angle $\vartheta_{l}$ with respect to the radar look-angle ϑ, and a random rotation β of the local incidence plane around the Line-Of-Sight (LOS) (see Figure 2), as stated by the following equations [19]:

(1) $$\{\begin{array}{c} \cos\vartheta_{l}=\frac{\cos\vartheta +b\sin\vartheta }{\sqrt{1+a^{2}+b^{2}}}\\ \tan\beta =\frac{a}{\sin\vartheta -b\cos\vartheta } \end{array}$$

where a and b represent, respectively, the facets’ slopes along the azimuth and range directions, which are modeled as independent Gaussian random processes.

Therefore, the incoherent first order backscattered electric field in the *H* (horizontal) and *V* (vertical) polarizations, namely ${\underline{E}}^{s}={\left(E_{H}^{s},E_{V}^{s}\right)}^{T}$, that arises from a generic randomly tilted facet can be expressed in terms of the incident field ${\underline{E}}^{i}={\left(E_{H}^{i},E_{V}^{i}\right)}^{T}$ as:

(2) $${\underline{E}}^{s}=\underline{\underline{S}}\cdot {\underline{E}}^{i}{\frac{e}{\pi r}}^{-jkr}$$

via the following scattering matrix [19,23]:

(3) $$\begin{array}{cc} \underline{\underline{S}}=\left(\begin{array}{cc} S_{HH} & S_{HV}\\ S_{HV} & S_{VV} \end{array}\right) & =k^{2}cos^{2}\vartheta_{l}\;I^{\zeta }\left(\vartheta_{l}\;,\beta \right)\left(\begin{array}{cc} \cos\beta & \sin\beta \\ -\sin\beta & \cos\beta \end{array}\right)\cdot \left(\begin{array}{cc} F_{H}\left(\varepsilon ,\vartheta_{l}\right) & 0\\ 0 & F_{V}\left(\varepsilon ,\vartheta_{l}\right) \end{array}\right)\\ & \cdot (\begin{array}{cc} \cos\beta & -\sin\beta \\ \sin\beta & \cos\beta \end{array}) \end{array}$$

where k is the wave number, *r* is the radar-to-target distance, $I^{\zeta }\left(\vartheta_{l}\;,\beta \right)$ is a polarization-independent function depending on the small-scale roughness ζ, while $F_{H}\left(\varepsilon ,\vartheta_{l}\right)$ and $F_{V}\left(\varepsilon ,\vartheta_{l}\right)$ represent the Bragg (SPM) or Fresnel (PO) coefficients for the *H* and *V* polarizations, with ε being the soil relative permittivity [19]. As Equation (3) shows, reciprocity (i.e., $S_{HV}=S_{VH}$) is assumed for the PTSM data analysis.

According to Equations (2) and (3), the second order statistics of the EM field backscattered from the generic tilted facet can be obtained by averaging the quantities $S_{pq}S_{uw}^{*}$ with respect to the small-scale roughness, where each of the indexes *p*, *q*, *u*, *w* can stand for the polarization *H* or *V*.

Moreover, assuming that the facets’ sizes are greater than both the EM wavelength and the correlation length of the small-scale roughness, but smaller than both the sensor geometric resolution and the correlation length of the large-scale roughness, it follows that the EM returns from different facets are uncorrelated. Accordingly, the second order statistics of the EM field backscattered from the whole surface can be obtained by averaging those of a single facet over $\vartheta_{l}$ and β or, equivalently, due to Equation (1), over the slopes a and b.

In particular, the elements of the polarimetric covariance matrix, that is, the powers $\sigma_{pq}$ and correlations $\Gamma_{pquw}$ of the polarimetric channels for the whole scattering surface, are:

(4) $$\left\{ \begin{array}{c} \sigma_{pq}={\langle \langle |S_{pq}|}^{2}\rangle_{{|}_{\zeta }}\rangle_{{|}_{a,b}}\\ \Gamma_{pquw}=\langle \langle S_{pq}S_{uw}^{*}\rangle_{{|}_{\zeta }}\rangle_{{|}_{a,b}} \end{array}\right.$$

where the operator $\langle \cdot \rangle_{{|}_{\psi }}$ represents the statistical mean with respect to the random variable ψ. Unfortunately, the statistical averages over a and b in Equation (4) cannot be computed in closed form [19,24]. However, assuming small values for the standard deviation σ of the facet slopes, a second order Taylor expansion of the quantities $\langle S_{pq}S_{uw}^{*}\rangle {}_{{|}_{\zeta }}$ around the mean slopes can be considered, thus leading to the PTSM solution for the EM field. In this regard, it is worth recalling that the mean slopes are tied to the topography of the considered surface, which in turn can be obtained by exploiting Digital Elevation Models.

The full expressions of the PTSM solution for the quantities in Equation (4), as well as their validity limits and the direct model validation, can be found in [2,19,23,24]. Here, we just want to stress that the PTSM is the only analytical model employed in the literature for soil moisture retrieval from PolSAR data, for which the statistics of the scattered field and, in particular, its depolarization and cross-polarization, come straightly from the stochastic properties of the scattering surface.

### 2.2. Retrieval Method Based on Co-Pol, Cross-Pol Ratios and Co-Polar Correlation Coefficient

The PTSM expressions for the covariance matrix elements of previous section depend on a number of parameters, whether characterizing the employed radar system (e.g., the operating frequency and the radar look-angle), or describing the geometrical (e.g., the large- and small-scale roughness) and physical (e.g., the soil permittivity) properties of the scattering surface. Among these, the latter are typically unknown, but can be retrieved from polarimetric radar data by adopting a well-posed inversion procedure.

**Figure 3:**
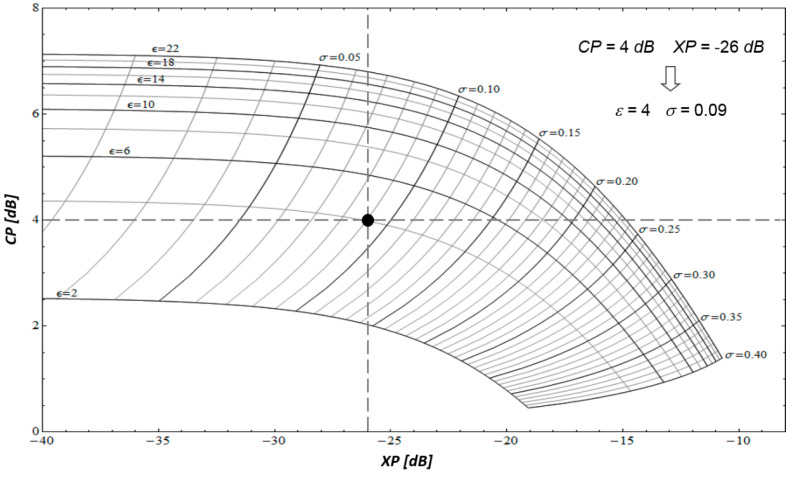
Co-pol/Cross-pol chart (radar look-angle equal to 45°) based on the PTSM approach and its usage for the soil permittivity and large-scale roughness retrieval: measured co-pol and cross-pol ratios equal to 4 dB and −24 dB, respectively, lead to soil permittivity and soil roughness estimates equal to 4 and 0.09, respectively.

To this aim, in [19,23,24] it has been shown that the co-polar (*CP*) and cross-polar (*XP*) ratios, as well as the co-polar correlation coefficient (γ), namely:

(5) $$\{\begin{array}{c} CP=\sigma_{VV}/\sigma_{HH}\\ XP=\sigma_{HV}/\sigma_{VV}\\ \gamma =\left|\Gamma_{HHVV}\right|/\sqrt{\sigma_{HH}\sigma_{VV}} \end{array}$$

are substantially insensitive to the small-scale roughness since, for fixed values of the radar look-angle and operating frequency, they only depend on the large-scale roughness σ and on the soil relative permittivity ε. Accordingly, it is possible to build up numerical charts where co-pol and cross-pol loci or co-pol and correlation coefficient loci are drowned for different values of σ and ε. Therefore, these charts, an example of which is depicted in Figure 3, can be used to retrieve the soil permittivity (and then, the soil moisture content [23]) and the large-scale roughness from measured co- and cross-pol ratios, or from measured co-pol ratios and co-polar correlation coefficients, which can be obtained, for instance, using polarimetric SAR images. In this regard, it has been shown that for bare soil surfaces exhibiting an anisotropic roughness (i.e., different roughness characteristics along the azimuth and range directions, as is the case, for instance, for tilled soils), the co-pol/correlation method outperforms to co-pol/cross-pol one for the estimation of ε (using a mixing model [26] for soil moisture) and σ [27].

PTSM-based retrieval methods have been applied and/or validated for P-, L- and X-band SAR data sets acquired over bare soils under various radar look-angles, and for different surface roughness, and soil moisture values (see for instance [2,16,19,23,24,25]). The best results are obtained at lower frequencies (P and L bands), which are less influenced by vegetation [16,17,18,19,20,21,22,23,24,25,26].

### 2.3. EM Scattering from a Slightly Vegetated Soil: The PTSTCM

**Figure 4:**
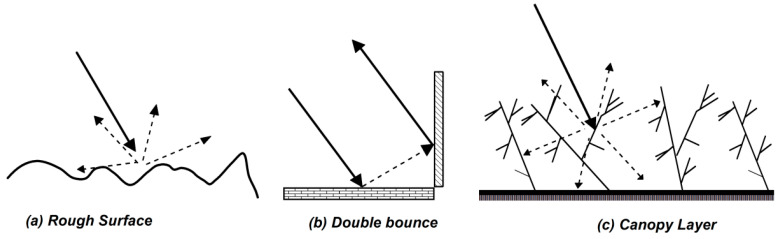
Surface (**a**), double-bounce (**b**) and volume (**c**) scattering mechanisms of electromagnetic (EM) waves.

What shown in Section 2.1 and Section 2.2 allows us to analytically predict the EM scattering from a bare soil surface and to make use of these predictions to retrieve soil surface parameters from polarimetric radar data. However, in many situations of practical interest, radar data are acquired over vegetated soils. In these contexts, the surface backscattering contribution is mixed with the radar returns arising from the vegetation cover, which in turn can be modeled as the superposition of double-bounce (i.e., ground-trunks interactions) and volume (i.e., canopy) scattering contributions (see Figure 4) [20]. Therefore, in such scenarios, a single-component model for the prediction of the EM backscattering, and its usage for parameters estimation, is shown to be ill-suited [19]. On the other hand, accounting for all the above-mentioned scattering components for parameters inversion purposes would require some quantitative a-priori knowledge about the characteristics of the observed scene (e.g., the value of the standard deviation of the soil roughness), as to devise well-posed inversion procedures [18,25].

However, in absence of any a-priori quantitative information, and assuming that the polarimetric radar data are acquired over slightly vegetated soils, where the ground-trunks interactions can be considered negligible, it is possible to resort to the PTSTCM [2]. This model is indeed founded on a two-component approach, where the scattered field is modeled as the superposition of independent surface and volume scattering contributions. In particular, the former is modeled by using the PTSM, via the scattering matrix and field statistics provided in Section 2.1. As for the latter, instead, it is modeled by a cloud of randomly oriented cylinder-like dipoles, whose scattering matrix is [20]:

(6) $$\tilde{\underline{\underline{S}}}=\left(\begin{array}{cc} \cos\varphi & \sin\varphi \\ -\sin\varphi & \cos\varphi \end{array}\right)\cdot \left(\begin{array}{cc} 0 & 0\\ 0 & \alpha_{v} \end{array}\right)\cdot \left(\begin{array}{cc} \cos\varphi & -\sin\varphi \\ \sin\varphi & \cos\varphi \end{array}\right)$$

where φ is the dipoles’ random orientation angle with respect to the vertical polarization direction, and $\alpha_{v}$ is a function of parameters describing the dipole cloud. Accordingly, the second order statistics for the backscattering from the canopy layer can be expressed as:

(7) $$\{\begin{array}{c} {\tilde{\sigma }}_{pq}={\langle |{\tilde{S}}_{pq}|}^{2}\rangle_{{|}_{\varphi }}=\delta_{pq}f_{v}\\ {\tilde{\Gamma }}_{pquw}=\langle {\tilde{S}}_{pq}{\tilde{S}}_{uw}^{*}\rangle_{{|}_{\varphi }}=\rho_{pquw}f_{v} \end{array}$$

where $f_{v}={\left|\alpha_{v}\right|}^{2}$, while the $\delta_{pq}$ and $\rho_{pquw}$ coefficients only depend on the probability density function p(φ) of the dipoles’ orientation φ. In particular, the value of these coefficients for three different probability density functions, corresponding to uniform dipoles orientation, prevalently horizontally distributed dipoles and prevalently vertically distributed dipoles, can be found in [2].

Finally, due to the independence between the surface and volume scattering mechanisms, the overall polarimetric powers $\sigma_{pq}^{tot}$ and correlations $\Gamma_{pquw}^{tot}$ relevant to the slightly vegetated soils can be expressed as the sum of the quantities in Equations (4) and (7).

### 2.4. Retrieval Method Based on the Modified Co-Pol Ratio and Co-Polar Correlation Coefficient

In Section 2.2, it has been shown that a proper combination of the elements of the covariance matrix in Equation (4) allows us to build up a well-posed retrieval procedure to get both the soil roughness and permittivity from polarimetric radar data acquired over a bare soil.

Similarly, in this section, we resume the methodology proposed in [2] to retrieve the soil roughness and permittivity from radar data acquired over a slightly vegetated soil. In particular, the following modified co-pol ratio and co-polar correlation coefficient:

(8) $$\{\begin{array}{c} CP_{mod}=\frac{\sigma_{VV}^{tot}-\frac{\delta_{VV}}{\delta_{HV}}\sigma_{HV}^{tot}}{\sigma_{HH}^{tot}-\frac{\delta_{HH}}{\delta_{HV}}\sigma_{HV}^{tot}}\\ \gamma_{mod}=\frac{\left|\Gamma_{HHVV}^{tot}-\frac{\rho_{HHVV}}{\delta_{HV}}\sigma_{HV}^{tot}\right|}{\sqrt{\left(\sigma_{HH}^{tot}-\frac{\delta_{HH}}{\delta_{HV}}\sigma_{HV}^{tot}\right)\left(\sigma_{VV}^{tot}-\frac{\delta_{VV}}{\delta_{HV}}\sigma_{HV}^{tot}\right)}} \end{array}$$

are only related to the surface parameters ε and σ, since the volumetric contribution $f_{v}$ cancels out.

Accordingly, also in this case, it is possible to build up numerical charts where modified co-pol ratio and correlation coefficient loci are drowned for different values of σ and ε. These charts can then be used to retrieve the soil permittivity (and then the soil moisture content) and roughness from polarimetric radar data. The method has been tested with L-band SAR data acquired over natural fields characterized by different phenology, and during a period covering all the phases of the vegetation growth. From such an analysis, it turned out that, as already mentioned, in presence of low vegetation (vegetation height lower than 50 cm, or cross-polarized ratio smaller than 0.1, and negligible double-bounce component), the PTSTCM soil moisture estimates were in better agreement with in situ measurements than the ones provided by the retrieval method based on the IGHD [2].

### 2.5. The Hallikainen’s Mixing Model of Volumetric Soil Moisture Retrieval from Permittivity

The Hallikainen’s mixing model has been employed to retrieve volumetric soil moisture from permittivity [26]. This mixing model was developed exploiting data of five soil types with wide textural (geotechnical) compositions, and employing nominal frequencies of 1.4, 4, 6, 8, 10, 12, 14, 16 and 18 GHz [26]. In the Hallikainen’s mixing model, the real and imaginary parts of the (complex) permittivity are second order polynomial functions of the volumetric soil moisture, whose coefficients depend on the sand and clay textural components of the soil. The general form of the Hallikainen’s mixing equation reads:

(9) $$\{\begin{array}{c} \varepsilon =a_{0}+a_{1}\times SAND+a_{2}\times CLAY+\left(b_{0}+b_{1}\times SAND+b_{2}\times CLAY\right)m_{v}\\ +\left(c_{0}+c_{1}\times SAND+c_{2}\times CLAY\right)m_{v}{}^{2} \end{array}$$

where *SAND* and *CLAY* are sand and clay textural components in percent by weight, and $a_{i},\;b_{i},\;\mathrm{and}\;c_{i}$ are the function of the (given) frequency, with their values are given in [26].

## 3. Software Design and Implementation

PTSM-OOP from the University of Naples Federico II is a software tool developed in the IDL environment, aimed at retrieving soil surface parameters from polarimetric SAR data. PTSM-OOP is an open source project available for public usage via [28] under *course material*.

We chose IDL for several reasons. First of all, it allows to easily manage large images, such as remote sensing SAR ones. In addition, with almost no required change in the source code, it can be run both on Windows and Linux systems. Finally, IDL is the basic language behind ENVI, so that our software can be easily employed in conjunction with ENVI.

**Figure 5:**
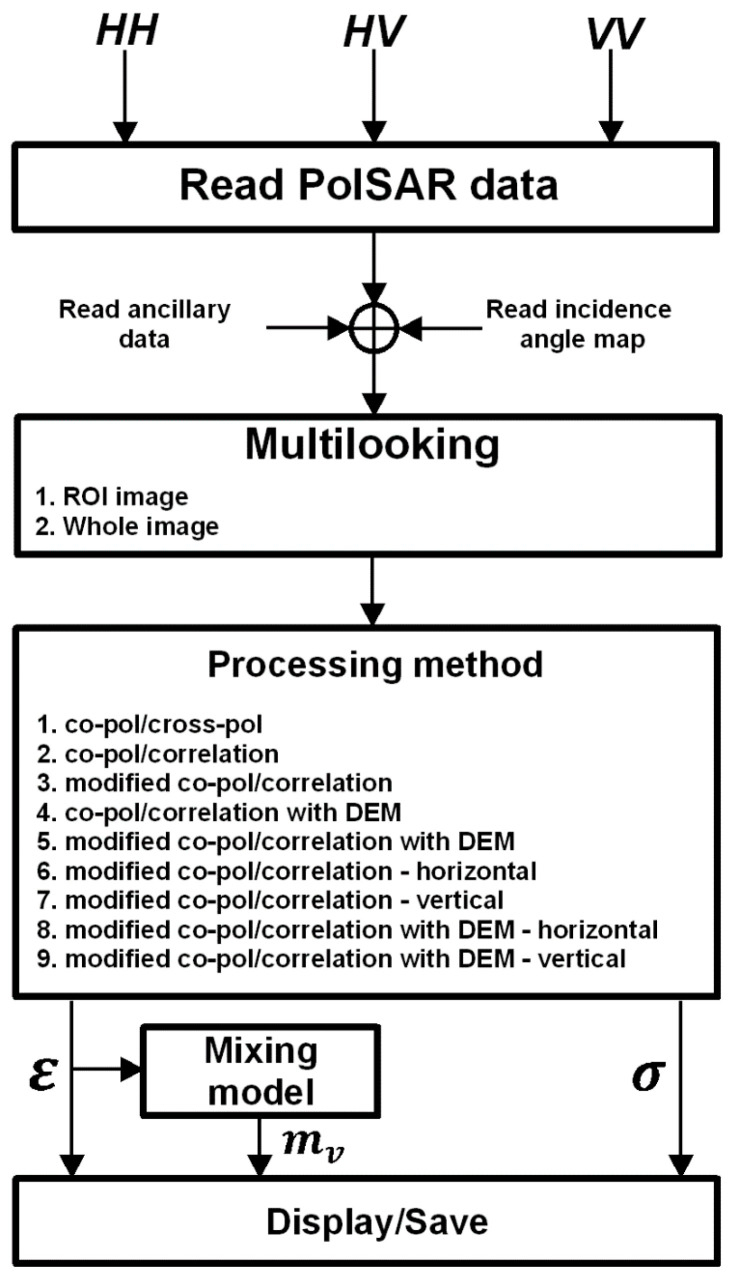
PTSM-Object-Oriented Program (OOP) program workflow, from the reading of polarimetric Synthetic Aperture Radar (SAR) data, to the visualization of permittivity ε, surface roughness σ and percentage of volumetric water content m_v_. ROI: Region of Interest.

A freely available IDL virtual machine is needed to run the software. The overall PTSM-OOP workflow is depicted in the block scheme of Figure 5 and it will be described in the following.

**Figure 6:**
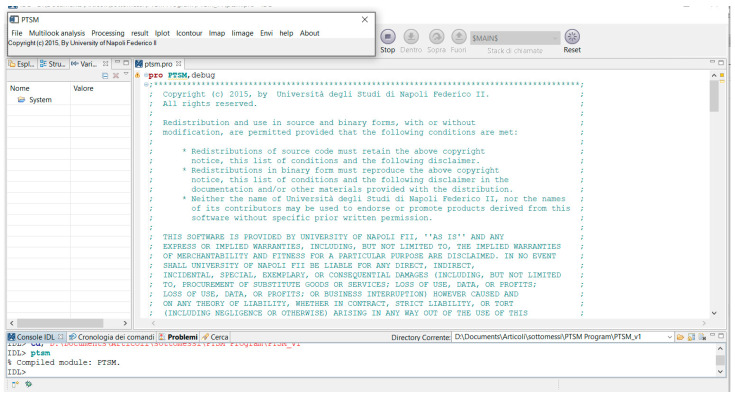
PTSM-OOP’s main window and copyright statements.

The user provides the PolSAR data to the software and gets as output the maps of soil moisture, soil permittivity and large-scale roughness. The IDL compiler checks the source codes that are selected by the user, and then applies the proper algorithm. PTSM-OOP Graphical User Interface (GUI) has menu entries in the main bar (see top of Figure 6) to access all application’s functionality. The user can read PolSAR data channels (i.e., *HH*, *VV* and *HV* complex data), ancillary file (including line and column numbers and spacings), and incidence-angle maps (that are particularly useful for scenarios with significant topography and/or for airborne SAR acquisitions, where the radar look angle significantly changes within the radar swath) in the “file” menu. Further details on input data will be provided in Section 4.

The “exit” menu item of the “file” menu allows to exit from the application.

After reading the PolSAR data and the ancillary information in the “file” menu, the next step is the multi-look analysis. Here, the user can select between two options, namely “Whole image multi-looking mode” and “ROI image multi-looking mode,” depending on whether the entire image or a selected Region of Interest (ROI) has to be considered. “Multi-looking” operation computes powers of the three polarimetric channels by averaging the square modulus of each channel over rectangular windows of size 10 by *n*, where *n* is such that the final pixel is approximately square. In addition, the copol correlation is computed by averaging in the same way the product of *HH* by the complex conjugate of *VV*. These operations also allow to reduce the effect of speckle. After this, it follows the core of the software tool, namely the “Processing” step. Here, the user can select among nine possible modes from the menu, according to the desired approach for the soil moisture and roughness retrieval. These nine approaches are described in the following section.

### 3.1. Co- and Cross-Polarized Ratios (Co-Pol/Cross-Pol) Method

If this option is selected, the retrieval method described in [19], and briefly recalled in Section 2.2, is employed. From the input measured SAR polarimetric data, $\sigma_{HH}$, $\sigma_{HV}$, $\sigma_{VV}$ are evaluated and the co-polarized and cross-polarized ratios (i.e., Equation (5)) are computed. For each (multi-look) image pixel, they are then compared to the charts of co-polarized and cross-polarized ratios computed from the model as a function of dielectric constant ε and large-scale rms slope σ (Figure 3), and the best fit pair of above-mentioned parameters is selected. Finally, the retrieved dielectric constant is converted into volumetric soil moisture $m_{V}$ using the Hallikainen model (Section 2.5). This approach can be selected if the analyzed scenario is a bare soil in a flat area.

### 3.2. Co-Polarized Ratio and Correlation Coefficient (Co-Pol/Correlation) Method

In this option, the initial settings and retrieval rationale are the same as those of Section 3.1, but the co-polarized ratio and the co-polarized correlation coefficient are exploited for the inversion, according to the approach described in [24]. Just as the previous one, this approach can be safely used only if the analyzed scenario is a bare soil in a flat area. However, it has the advantage that only co-polarized channels *HH* and *VV* are needed (dual-pol). In addition, recent studies [27] show that this approach is more robust to surface roughness anisotropy than the previous one. Therefore, it must be preferred if a tilled bare soil is considered.

### 3.3. Modified Co-Polarized Ratio and Correlation Coefficient (Modified Co-Pol/Correlation) Method

This option is founded on PTSTCM approach summarized in Section 2.3 and Section 2.4, in which the volumetric scattering contribution from the (moderate) vegetation cover is described via a dipole-cloud model with a uniform distribution of the dipole orientation angle [2]. This option can be selected if the considered scenario is a moderately vegetated soil in a flat area, and no specific a priori information on vegetation type is available.

### 3.4. Co-Pol/Correlation Method with DEM

This approach is basically the same as the one of Section 3.2, extended to account for the local surface non-zero mean slopes related to macroscopic topography, according to the approach described in [23,24]. In this case, a Digital Elevation Model (DEM) must also be provided as input. The latter is used for the calculation of the local (i.e., at the pixel scale) mean slopes. This approach can be selected if the analyzed scenario is a bare soil in the presence of non-negligible topography.

### 3.5. Modified Co-Pol/Correlation Method with DEM

This approach is basically the same as the one of Section 3.3, extended to account for the local surface non-zero mean slopes related to macroscopic topography, according to the approach described in [23,24]. In this case, also a DEM must be provided as input, see Section 3.4. This approach can be selected if the analyzed scenario is a moderately vegetated soil in the presence of non-negligible topography, and no specific a priori information on vegetation type is available.

### 3.6. Modified Co-Pol/Correlation Method—Horizontal

This approach is basically the same as the one of Section 3.3, but in this case the volumetric scattering contribution from (moderate) vegetation is described via a dipole-cloud model with prevalently horizontal dipole orientation angle [2]. This option can be selected if the considered scenario is a moderately vegetated soil in a flat area, and available a priori information on vegetation type leads to assume a prevalently horizontal distribution of leaves and/or branches.

### 3.7. Modified Co-Pol/Correlation Method—Vertical

This approach is the same as the one in Section 3.6, but with a dipole-cloud model with prevalently vertical dipole orientation angle.

### 3.8. Modified Co-Pol/Correlation Method with DEM—Horizontal

This option is the same as the one of Section 3.6, but it requires an input DEM to account for macroscopic topography. This approach can be selected if the analyzed scenario is a moderately vegetated soil in the presence of non-negligible topography, and available a priori information on vegetation type leads to assume a prevalently horizontal distribution of leaves and/or branches.

### 3.9. Modified Co-Pol/Correlation Method with DEM—Vertical

This option is the same as the one of Section 3.8, but with a dipole-cloud model with prevalently vertical dipole orientation angle.

Finally, the “Result” menu allows the user selecting the desired output map. In the first option the map of dielectric constant ε is shown; in the second option the map of the large-scale roughness is shown; finally, in the last option, the map of the volumetric water content is provided to the user. Since the software is written in IDL environment, the output images will be given in *.png, *.JPEG, *.tiff, *.PDF, and similar formats. They are also automatically stored in .dat files in floating point format, for possible further analysis.

## 4. Program Functionality

GUI is a point-and-click way of running an application in a windowed environment. Complex and repetitive tasks can be simplified by an interactive GUI, which hides the function and implementation details and allows the user to focus on the data, rather than on the mechanism of entering IDL commands to the program itself. PTSM-OOP GUI environment is provided in IDL programming language. Another advantage of PTSM-OOP GUI toolkit is the ability to be run on different platforms, without changing the appearance of the mapping GUI.

**Figure 7:**
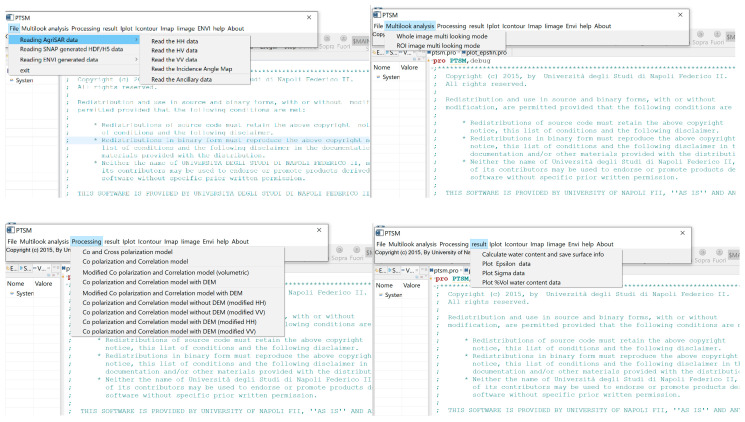
PTSM-OOP’s scrolling list for File, Multilook analysis, Processing and Results.

PTSM-OOP GUI application contains a number of components known as widgets, each of which having a specific appearance. In particular, PTSM-OOP GUI has the following widgets:

1. A menu bar (main bar), which allows the configuration of application properties.
2. Scrolling lists, which enable the selection of input data, processing method and output map to display (Figure 7).
3. IDL graphical windows displaying the output imageries.

PTSM-OOP application responds to any interaction with each widget in a unique way. When user’s interaction with the PTSM-OOP GUI is detected, the PTSM-OOP GUI takes particular course of action depending on the selected widgets. The main steps of the input procedure are guided, and the *catch error* function provides the control on the possible user errors. The execution time for each PTSM-OOP run depends on the size of the input images (i.e., *HH*, *VV* and *HV* layers).

The PTSM-OOP’s functions are listed below:

1. A base widget named PTSM which has the form of widget_base(). This widget contains all the widgets subsequently created inside the GUI. This type of the widget is known as top-level base widget, which is a parent to all the child widgets contained in the PTSM-OOP GUI.
2. A widget_label(), creates a label widget and returns the widget identifier label.
3. Number of widget_button(), have control of the GUIs buttons.
4. Finally, a widget_control(), which is called to render on screen the widget hierarchy controlled by base_ widget().

Widget buttons needed to read input data are under the “file” button. Before mentioning them, it is useful to describe the required format of input data for the subsequent “multi-looking” step. Each polarimetric channel (which is a complex image) must be a two-dimensional array of complex numbers, stored in an *.dat file. The number of lines and columns, and line and column spacings, must be provided as a simple text file (ancillary data). Finally, a two-dimensional array of real numbers, with the same size of SAR data, containing the incidence angle map, stored in an *.dat file (incidence-angle map file), can be optionally used. Therefore, a preprocessing step aimed at converting the input PolSAR data in this format is required. Of course, this step strongly depends on the storage format of the considered dataset: the PTSM-OOP software can perform this preprocessing step, with no need for external tools, when the original SAR data are in *.h5 (HDF) format or if they are E-SAR data of the AGRISAR mission [29]. Otherwise, the user can use the freely available ESA SNAP package to generate HDF format data from original SAR data of the main polarimetric sensors, such as ALOS, RADARSAT, etc. Alternatively, preprocessing can be carried out with most of the licensed (e.g., ENVI, IDL, Matlab) or free (e.g., SNAP, PolSAR PRO) packages that are usually employed to process SAR data. For instance, since IDL is connected to ENVI software, all the formats that can be read by ENVI can be transformed to the required *.dat format via *Export to IDL Variable*, which is a built-in function in ENVI.

In conclusion, three widget_buttons read the input data under the “file” -> reading data button. They are named: Read_the_HH_data, Read_the_HV_data, Read_the_VV_data, Read_the_incidence angle map, Read_the_ancillary data and PTSM_exit.

In the “Multilook analysis” button, two widget_buttons, which are named *Whole_image_multilooking_mode* and *ROI_image_multilooking_mode*, are handling the Multilooking step.

In the “Processing” button, nine widget_buttons allow the selection among the processing methods listed in Figure 5 and described in Section 3.

In the “Result” button of PTSM-OOP software, four widget_buttons exist. They are named Calculate_water_content_and_save_surface_info, Plot_epsilon_data, Plot_sigma_data and Plot_%volumetric_water_content_data.

In each of the remaining buttons (Iplot, Icontour, Imap, Iimage, ENVI, Help, About), only one widget_button is acting. They are designed to call functions from ENVI software, and to display HELP/About information.

## 5. Program Run Example

**Figure 8:**
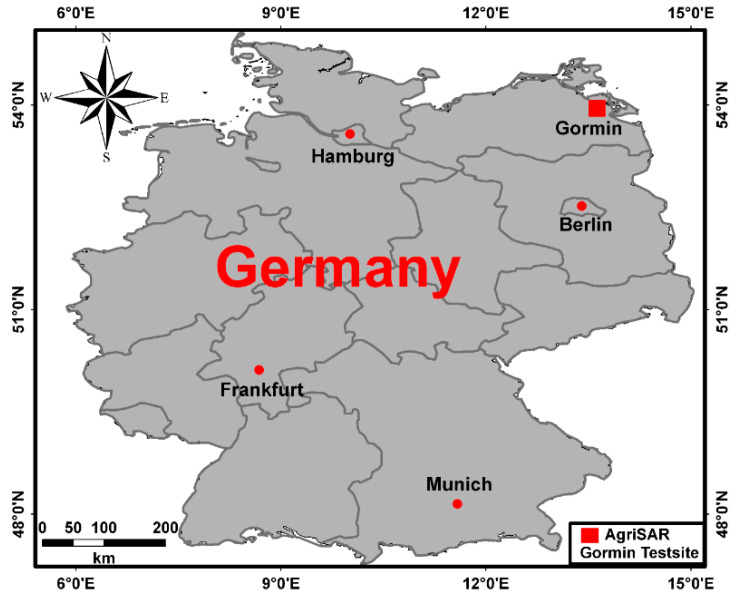
Location of the Gormin farm Test-site (red rectangle) in German federal state Mecklenburg-Western Pomerania.

In this section we illustrate a step by step example procedure of PTSM-OOP, to obtain meaningful soil parameters from fully polarimetric SAR images. The employed SAR data were acquired in the framework of the 2006 AgriSAR campaign (Figure 8) [29]. The campaign was funded by the European Space Agency (ESA) with the main goal of collecting in-situ measurements, microwave and optical remote sensing data over an intensive farming region in Demmin (Germany), during an entire vegetation growth period. DEMMIN (Durable Environmental Multidisciplinary Monitoring Network) is a well-known test site located in Mecklenburg-Western Pomerania North-East Germany, approximately 60 km north of Neustrelitz and 150 km north of Berlin (Figure 8). The general information about the E-SAR data are given in Table 1. In particular, PolSAR data were acquired by the E-SAR (Experimental-SAR) system, a polarimetric airborne SAR sensor operated by the German Aerospace Center (DLR), from 1988 until November 2009.

The sensor operated at three different bands/channels (X, C and L), and it was mounted onboard a Dornier Do-228 aircraft, operating in a range of flight altitudes between 300 m and 6000 m above ground. For each flight the sensor was able to collect data at a single center frequency (i.e., no simultaneous multi-bands acquisitions were possible), either with one, two, or four polarization channels. However, the system was enabled to get fully polarimetric acquisitions only at L-band. Data were freely delivered to the scientific community at two processing levels, namely the Radar Geometry Image product (RGI) and the Geo-coded and Terrain-Corrected product (GTC).

RGI (SLC) products exhibits a slant range resolution equal to 2 m and an azimuth resolution equal to {0.9 m, 0.9 m, 1 m} (for X-, C- and L-band, respectively), while a 2 m × 2 m grid in WGS-84 UTM projection, zone 33, has been used for GTC products [29].

In particular, in this study retrieval of soil surface parameters via PTSM-OOP algorithm was applied on the available Single Look Complex (SLC) geo-coded L-band quad-polarimetric GTC SAR images.

**Figure 9:**
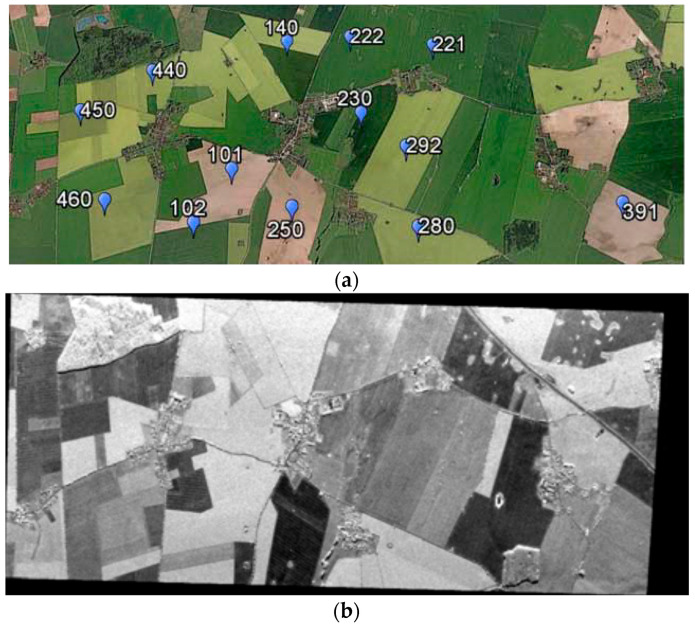
(**a**) Optical image of the Demmin study area, Germany, with indication of in situ measurements. (**b**) E-SAR HH-pol image. North is the top of the image, east is on the right. The area is about 8500 m × 3000 m wide.

The study area is characterized by several crop types, such as: sugar beet, wheat, winter barley, winter rape, grassland and corn. Optical and SAR images of the area are shown in Figure 9. A wide range of ground truth data collected at the same time of the radar acquisitions are available: vegetation phenology, terrain conditions, precipitations, and volumetric soil moisture. In particular, the last was measured with different techniques (i.e., time-domain reflectometry, gravimetric and capacitive measurements) and different time-sampling scenarios (intensive campaigns over many fields, see Figure 9a, weekly measures on a limited set of fields and via continuous measurements stations over few fields) [29]. All details on the in-situ measurement campaign are reported in [29].

AGRISAR data (including both SAR data and ground truth) can be received free of charge from ESA by following the procedure described in [30].

### 5.1. Read the Input Data

The PTSM-OOP procedure is started by reading the input PolSAR data including *HH, HV* and *VV* SAR channels/bands, incidence angle map of the study area and ancillary data of the available PolSAR images, via the “file” button of the PTSM-OOP program (Figure 7, top-left). The used data is named “06agrsar0609 × 1_t01” [29], and is part of 2006 AgriSAR campaign. “i06agrsar0609 × 1_ch1_t01_int_slc_geo.dat” (*HH*), “i06agrsar0609x1_ch2_t01_int_slc_geo.dat” (*HV*) and “i06agrsar0609x1_ch3_t01_int_slc_geo.dat” (*VV*) are the three PolSAR bands of the Demmin study area. “incmap06agrsar0609x1_t01.dat” is the given incidence angle map; “06agrsar0609x1_t01_README_GTC” is the ancillary data, which provides some information about the E-SAR sensor such as description of the campaign, SAR processing parameters file, repeat pass master identifier, DEM slant range, range pixel positions, pixel spacing north, minimum/maximum easting/northing etc. The E-SAR data are in the complex format. The processing parameters of E-SAR data are given in Table 1, including original pixel spacing; we considered a further multi-looking to the entire image in the software run, as described below.

### 5.2. Multi-Looking the Input Data

After reading the data to PTSM-OOP software, we multi-look the given PolSAR imageries as has been shown in Figure 7, top-right. In this step, powers and correlation are also computed according to Equation (4) by averaging over rectangular windows, and, accordingly, measured co-polar and cross-polar ratios, and co-polar correlation coefficients are computed via Equation (5) [19,24]. Multi-looking can be carried out in two different sections, depending on the necessity: (a) whole image multi-looking mode and (b) ROI multi-looking mode. In the given example, we use whole image multi-looking mode, and the employed window size is 10 by 10 pixels, so that the final map resolution is 20 m by 20 m. The incidence angle data also is resampled accordingly for further analysis. Depending on the necessities, ROI multi-looking mode also is available, in which the operator can select the regions which need to be processed.

### 5.3. Processing

After multi-looking analysis, we proceed to the processing step. The nine available processing modules are presented in the Section 3 and Figure 5. In this example, we use the “Co- and cross-polarized ratios (co-pol/cross-pol)” method (the first choice in the scrolling list) to retrieve the soil moisture parameters.

Theoretical values of Normal Radar Cross Sections (NRCS) for *HH, VV* and *HV* polarizations and of the *HHVV* correlation are computed for a set of values of *ε* and *σ*, as described briefly in Section 2.1, see Equation (4), and in more detail in [19] and [24]. Co-polar and cross-polar ratios are then computed via Equation (5). This results in a look-up table, which associates a co-polar and cross-polar ratios pair to each *ε*, *σ* pair (a graphical example of such a look-up table is provided by Figure 3).

The final step is the comparison of the measured co-polar and cross-polar ratios (i.e., those obtained from SAR data and computed in the multi-looking step) with the just-produced lookup tables to retrieve soil permittivity and soil roughness for each pixel.

The other eight processing modules follow analogous procedures, except that for methods of Section 3.4, Section 3.5, Section 3.8and Section 3.9, a file containing the surface DEM is requested as further input (for the considered case the surface is substantially flat and a DEM is not needed). This processing phase is completely unsupervised and no action is required to the user.

### 5.4. Results

**Figure 10:**
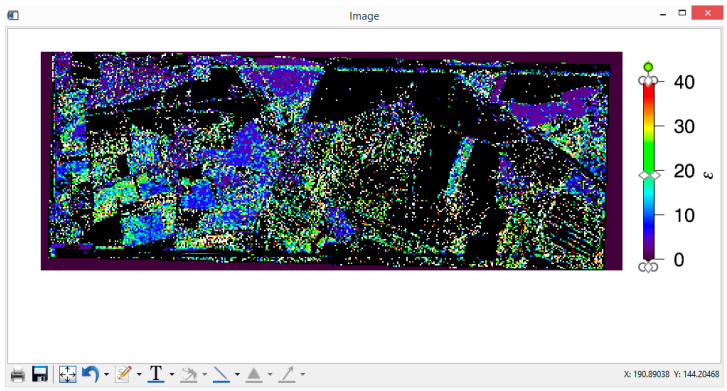
PTSM’s retrieval of dielectric constant ε for the Demmin area (16 May 2006). North is the top of the image, east is on the right.

**Figure 11:**
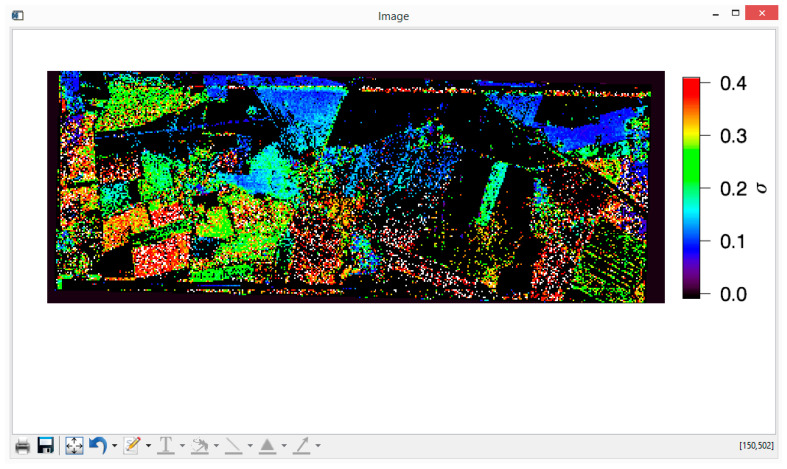
PTSM-OOP’s retrieval of large-scale roughness σ for the Demmin area (16 May 2006). North is the top of the image, east is on the right.

**Figure 12:**
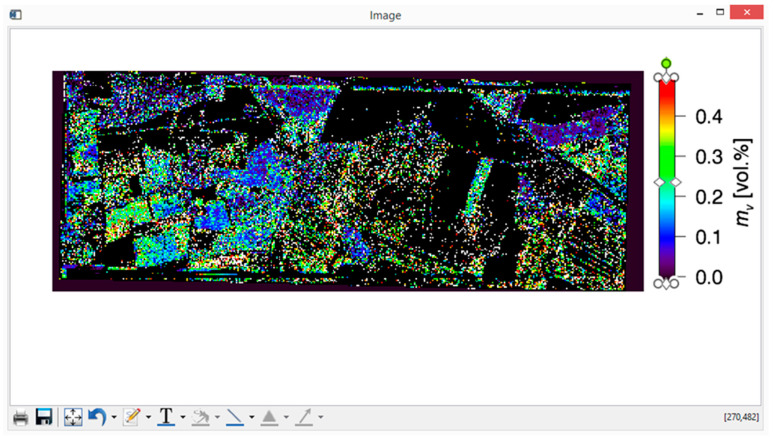
PTSM-OOP’s retrieval of volumetric soil moisture $m_{V}$ for the Demmin area (16 May 2006). North is the top of the image, east is on the right.

The only remaining task is the presentation of the results and calculation of (percentage of) water content for each given pixel. In Figure 10 and Figure 11, dielectric constant, ε, and large-scale roughness σ retrieval maps for Demmin study area for 16 May 2006, with co-pol/cross-pol method, are given. The color scale is showing the strength of the retrieval parameters (i.e., ε and σ), and the black pixels are corresponding to pixels for which no valid retrieval values are obtained. Conversion of dielectric constant ε to the (volumetric percentage of) water content is performed by using the Hallikainen mixing model of Section 2.5, with percentages of sand and clay of 68% and 7%, respectively [29]; in Figure 12, the soil moisture map obtained with this conversion is depicted for the Demmin area (16 May 2006). The generated images can be saved as ordinary image (or document) format files, such as *.png, *.JPEG, *.tiff, *.eps, *.PDF, etc. However, these maps are also automatically stored in .dat files in floating point format, for possible further analysis.

### 5.5. Comparison with Ground Truth Data

For the 16 May 2006 images, the vegetation average height is low, and, as stressed before, for low vegetation covers, PTSM (and/or PTSTCM) can be safely used [2]. In particular, by using the co-pol/cross-pol method, a root mean square error (RMSE) of 14 vol% and a percentage of invalid pixels of 31% are obtained; by using the modified copol/correlation method, RMSE and percentage of invalid pixels decrease to 9 vol% and 8%, respectively. These results are in agreement with what is expected considering the results in [2]. In both cases, the entire processing chain requires about 3 min for image size of about 4300 × 1600 pixels per polarimetric channel, on a laptop with an Intel I7 processor (a few minutes more are needed for ALOS and RADARSAT whole images).

A complete comparison of PTSM and PTSTCM results with ground truth data is available in the literature [2,16,19,23,24,25,27,28]. However, to further verify the correct implementation of these methods in the presented software, we carried out a similar analysis for the results of the software. In particular, we performed the same retrieval procedure described in the previous subsections for the polSAR acquisitions of 19 April 2006, 3 May 2006 and 11 May 2006, in order to consider the time period from 19 April to 16 May 2006, during which the vegetation was mostly in an early stage of growth and its average height was low (ranging from nearly zero, for corn fields, to more than 1 m, for rape fields [29]).

**Figure 13:**
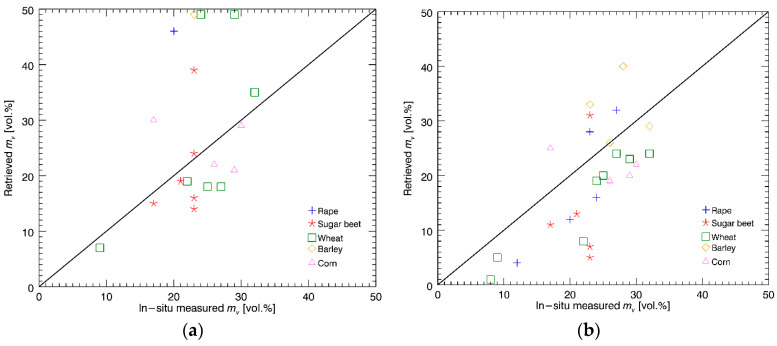
Scatterplot of the retrieval results vs. measured ground truth: (**a**) co-pol/correlation method; (**b**) modified co-pol/correlation method.

For each of these acquisitions, the overall results are similar to the ones obtained for the 16 May one (except for the 11 May acquisition, for which the results are slightly worse). In order to perform a more punctual comparison, we considered separately the soil moisture results for different fields. We underline that both SAR estimates and in situ measurements, relevant to each single field and for each single acquisition, show large standard deviations, often of the same order of mean values. Therefore, for each field and at each acquisition date, we compare average values of retrieved and in situ measured soil moisture. Results are presented in the scatterplots of Figure 13 for the co-pol/correlation method (a) and the modified co-pol/correlation method (b).

A visual analysis of these scatterplots shows that both methods provide reasonable results, but in the first one for some fields and acquisitions, a strong overestimate is obtained, whereas the second one, accounting for the effect of the (moderate) vegetation, is in better agreement with the ground truth. This is confirmed quantitatively by evaluating the correlation coefficient and RMSE for the two cases. In fact, for the standard co-pol/correlation method, the correlation coefficient is rather low, i.e., 0.34, and RMSE is 13.6 vol%, whereas for the modified co-pol/correlation method the correlation coefficient is 0.64, and RMSE is 9.1 vol%. In addition, these results are practically coincident with those obtained in [2] for the same considered time period, thus confirming the correct implementation of the methods in the software tool.

## 6. Conclusions

In this paper, a software tool for the automatic retrieval of soil surface parameters from SAR data and via PTSM and PTSTCM has been presented. The available methodologies for soil surface parameters retrieval have been presented, and the basic theory behind PTSM/PTSTCM has been briefly but thoroughly reviewed.

The presented software tool allows to create maps of dielectric constant ε, large-scale roughness σ, and volumetric soil moisture $m_{V}$ from fully polarimetric and/or dual polarimetric SAR images. In fact, PTSM can be also employed in the absence of cross-polarization (*HV*), if a bare soil is imaged. In this case, only co-pol ratios and *HH-VV* correlations are needed. Availability of fully polarimetric data allows obtaining realistic retrieved values also in moderately vegetated areas. The software tool implements nine different algorithms, to be selected according to the typology of the observed scenario, as specified in Section 3. Thanks to its user-friendly interface, the tool is suitable for both expert and non-expert users. With this respect, the presented tool bridges a gap in the available software tools for PolSAR processing: in fact, commercially and freely available software tools for the processing of SAR data that include routines for basic manipulation of polarimetric SAR data (e.g., coherency and covariance matrix calculation, Pauli decomposition, etc.) certainly exist, see Section 1, but they do not implement easy-to-use methods for surface parameter retrieval. This has been the main motivation of our work.

In order to provide a first guide to the use of the tool, its application to a specific example from Germany’s Demmin test-site, for which ground truth is available, has been presented. Finally, a comparison of retrieved soil moisture with in-situ measurements has been illustrated, showing a good agreement, at least for the method accounting for the presence of vegetation (modified co-pol/correlation). Obtained retrieval results are in agreement with what was expected according to the available literature [2].

The presented software is certainly amenable of further developments. For instance, use of compact polarimetric SAR data for soil moisture retrieval has been proposed [31]. At the moment, compact-pol SAR data cannot be employed by the software tool, since the PTSM and PTSTCM should be properly modified to cope with such data: this is left to future work.

## Figures and Tables

**Table 1:** E-SAR L-band radar parameters for Demmin scene.

SENSOR:	E-SAR
LOCATION:	Demmin
LONGITUDE-E (START OF FLIGHT TRACK) [deg]:	13.35661
LATITUDE-N (START OF FLIGHT TRACK) [deg]:	54.020437
ACQUISITION DATE:	16 May 2006
TIME OF START OF TRACK [UTC]:	17:41:25
AVERAGE TERRAIN ELEVATION [m]:	20
ALTITUDE ABOVE MEAN SEA LEVEL [m]:	3658.27
ANTENNA DEPRESSION ANGLE [deg]:	40
TRACK ANGLE TRUE [deg]:	−89.9996
RADAR CENTER FREQUENCY [GHz]:	1.3
POLARIZATION:	Fully Polarimetric
ADC-SAMPLING FREQUENCY [MHz]:	100
ONBOARD RANGE COMPRESSION:	OFF
RANGE DELAY OF THE FIRST RANGE BIN [us]:	22.72
PULSE REPETITION FREQUENCY (PRF) [Hz]:	1600
START TIME OF THE IMAGE:	17:41:31
END TIME OF THE IMAGE:	17:44:00
CALIBRATION SCALE FACTOR [dB]:	60
RANGE RESOLUTION [m]	2.12027
AZIMUTH RESOLUTION (SLC) [m]	1
RESOLUTION (DETECTED) [m]	2 × 2
LOOK OVERLAP [%]	50
NUMBER OF LOOKS	8
PROCESSED SQUINT ANGLE [deg]	−0.92715
AIRCRAFT GROUND SPEED [m/s]	89
NUMBER OF RANGE LINES SKIPPED	0
NUMBER OF RANGE LINES PROCESSED	237,345
